# Ischemia-modified albumin use as a prognostic factor in coronary bypass surgery

**DOI:** 10.1186/1749-8090-7-3

**Published:** 2012-01-05

**Authors:** Muhip Kanko, Sadan Yavuz, Can Duman, Tulay Hosten, Emin Oner, Turan Berki

**Affiliations:** 1 Kocaeli University Medical Faculty Department of Cardiovascular Surgery; 41380; Kocaeli, Turkey; 2 Canakkale Onsekiz Mart University Medical Faculty Department of Biochemistry; 17100, Canakkale, Turkey; 3 Kocaeli University Medical Faculty Department of Anaesthesiology; 41380, Kocaeli, Turkey

**Keywords:** Coronary artery bypass surgery, ischemia-modified albumine, myocardial ischaemia

## Abstract

**Background:**

Various types of markers have been used so far in order to reveal myocardial perfusion defect. However, these markers usually appear in the necrosis phase or in the late stage. Having been the focus of various investigations recently, ischemia-modified albumin (IMA) is helpful in establishing diagnosis in the early stages of ischemia, before necrosis develops.

**Methods and Results:**

30 patients that underwent only coronary bypass surgery due to ischemic heart disease within a specific period of time have been included in the study. IMA levels were studied in the preoperative, intraoperative, and postoperative periods. The albumin cobalt binding assay was used for IMA determination. Hemodynamic parameters (atrial fibrillation, the need for inotropic support, ventricular arrhythmia) of the patients in the postoperative stage were evaluated. Intraoperative measurement values (mean ± SD) of IMA (0.67677 ± 0.09985) were statistically significantly higher than those in the preoperative (0.81516 ± 0.08894) and postoperative (0.70477 ± 0.07523) measurements. Considering atrial fibrillation and need for inotropics, a parallelism was detected with the levels of IMA.

**Conclusions:**

IMA is an early-rising marker of cardiac ischemia and enables providing a direction for the treatment at early phases.

## Background

Recent studies show that the structure of serum albumin changes when ischemia develops in the body. From this point of view, studies focusing on a new marker for myocardial ischemia have been carried out. Ischemia Modified Albumin is a new marker used to detect myocardial ischemia and it shows an early change [1,2]. Elevations in IMA levels have been detected following percutaneous coronary interventions [3,4]. It is also used for differential diagnosis in patients admitting to emergency departments with angina pectoris [5]. It has recently been reported that IMA also increases in case of pulmonary infarction, critical limb ischemia, cerebrovascular disease conditions [6-10].

Ischemia-modified albumin (IMA), a Food and Drug Administration-approved serum biomarker of cardiac ischemia and a risk stratification tool for suspected acute coronary syndrome, is produced during an ischemic condition or attack and is present in the blood in early and easily detectable levels [5,11]. The increase in markers that predict myocyte necrosis during or after CABG surgery usually parallels with a poor prognosis of the patient. Numerous markers have been used to reveal myocardial necrosis until today [12,13]. These markers have been used as diagnostic criteria for myocardial necrosis that develops after CABG operations [14]. IMA is a marker formed after damage in the N terminal region of the albumin in ischemic conditions. Endothelial or extracellular hypoxia, acidosis, and free oxygen radicals have been shown to cause IMA increase [15]. In this study, we have intended to determine the association between IMA levels and hemodynamic parameters of the patients.

## Materials and methods

The study was planned upon obtaining approval from the ethics board of our hospital. 30 patients, on which merely CABG was to be performed within a certain time frame, were included in the study. Patients who required concurrent valve or another cardiac intervention were not included in the study. Patients with serious liver, kidney, peripheral vascular, or cerebral pathologies were also excluded from the study.

### Surgical procedure

Anesthetic management was uniform in all patients. Midazolam was used for premedication, and a combination of fentanyl, midazolam and pancuronium was used for the induction. After intubation, mechanical ventilation with oxygen and nitrogen was initiated. Anesthesia was maintained with midazolam, vecuronium bromide and inhaled sevoflurane. Patients were all operated on by the same surgical team. CABG was performed with moderate hypothermia. After the aorta was cross-clamped and 1000 mL of high-potassium cold-blood cardioplegic solution (24° to 26°C) (10-15 mL/kg) was perfused one time in an antegrade, myocardial revascularization was performed during cardioplegic arrest at 28°C to 30°C. The cardioplegic solution was composed of whole blood with a hemoglobin level of 8 g/dL and 25 mEq/L KCl. Following antegrade cardioplegia, cardioplegia solution containing 10 mEq/L KCl was constantly administered via retrograde path through the coronary sinus. Distal anastomoses were made. Proximal anastomoses were performed under side clamping. Following the completion of the surgery, the patients were taken to intensive care. Standard postoperative care was given to all the patients. Extubation was performed at the earliest stage possible following the provision of hemodynamic stability. The first choice when inotropic agent requirement emerged was dopamine. At times when atrial fibrillation occurred, amiodarone was the first choice for treatment in case laboratory and blood gas values were normal.

### Biochemical assays

Blood samples were drawn from the coronary sinus right before cross-clamping and 30 minutes after cross-clamping. Six hours after the operation, other blood samples were taken from a central venous catheter, which was forwarded into the right atrium and the IMA levels were studied. The blood samples were placed in flat tubes and kept waiting for 30 minutes, then sera were separated by centrifuging at 3000 rpm for 10 minutes. Then the obtained serum samples were subjected to measurement immediately. Albumin Cobalt binding test was analyzed according to the method defined by Bar-Or et al. [16]. In this method 200 mL serum was added to the water solution of 50 mL %0, 1 (w/v) cobalt chloridine (sigma;CoCl_2_.6H_2_O). It was mixed gently and waited for 10 minutes for sufficient cobalt-albumin binding. Then 50 mL dithiothreitol (DTT) (Sigma 1.5 mg/mL H_2_O) was added as a colorizing agent. After waiting for two minutes 1.0 mL 0.9% NaCl was added to stop the cobalt binding process of albumin. Afterwards, the absorbance was measured through spectrophotometer at 470 nm (Shimadzu, model UV160U). Sample blank without DTT were used as blind. The results were reported as absorbance units (ABSU).

### Statistical Analysis

The analyses were performed using SPSS for Windows, version 13.0. Normal distribution test was applied. Due to its preoperative variable that does not confirm to normal distribution, non-parametric tests were performed in comparisons containing the preoperative variable. Based on the normal distribution analysis of data, the Friedman test, Wilcoxon Signed Rank test and Paired sample t test were used in the comparisons of the dependent groups, while independent group comparisons were conducted using the Mann-Whitney U test. The level of statistical significance was taken as p < 0.05 and the Bonferroni correction was used where needed.

## Results

**Figure 1:**
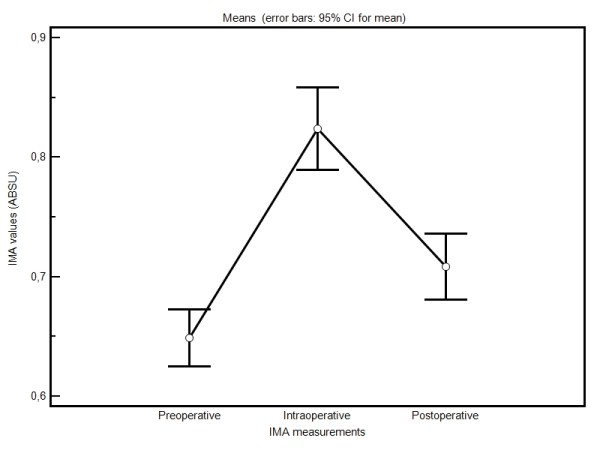
**Changes in all measurements of the cases**.

22 of the patients were males while 8 were females. The mean age of the patients was 58,6 (39 to 79). The demographics characteristics of the patients are given in the Table 1. In the echocardiograms of the cases, the ejection fractions were 35% and below in 12 cases (40%); between 36% and 50% in 7 cases (23%) and above 51% in 11 cases (37%). Single-vessel bypass surgery in one case, 2-vessel bypass surgery in another case, 3-vessel bypass surgery in 11 cases, 4-vessel bypass surgery in 14 cases, and 5-vessel bypass surgery in 3 cases were performed. LIMA was used as a graft for LAD bypass in a total of 27 cases. For other vessel bypasses, Vena saphena magna (the great saphenous vein) was used. One (3%) of the cases died. The mean cardiopulmonary bypass duration was 81 minutes and the mean cross-clamping duration was 37 minutes. In 23 (76%) patients, a need for a cardiotonic occurred while two (7%) cases required intra-aortic balloon. Cases in whom intra aortic balloon was needed were urgent patients who were taken to bypass surgery in a hemodynamically unstable condition. Arrhythmias not requiring treatment developed in 11 (36%) of the cases in the postoperative period. Paroxysmal AF attacks developed in 16 (53%) cases during the postoperative period and 6 of these cases needed treatment for more than 72 hours. All the cases were discharged from the hospital with sinusoidal rhythm. Preoperative, peroperative, and postoperative changes in IMA of the patients were compared, and it was observed that these changes were significantly different from each other (Friedman test, p < 0.05). Postoperative IMA measurement values were higher than those of preoperative IMA. There was a statistically significant difference between the preoperative and postoperative measurements (p = 0.011) (Wilcoxon signed rank test). In terms of preoperative IMA measurements and intraoperative measurements, the IMA levels at the intraoperative stage were higher. The difference between these values were statistically significant (p < 0.001) (Wilcoxon signed rank test). When comparing the intraoperative and postoperative measurements of IMA, intraoperative phase levels were higher again and the difference between them was statistically significant (p = 0.001) (Paired sample t test) (Figure 1). Arrhythmias not requiring treatment developed in 11 (36%) of the cases in the postoperative period. Paroxysmal AF attacks developed in 16 (53%) cases during the postoperative period and 6 of these cases needed treatment for more than 72 hours. All the cases were discharged from the hospital with sinusoidal rhythm. 23 (76%) of the cases required an inotropic (7-15 microgram/kg/min dopamine). The need for an inotropic disappeared in five of the cases within 6 hours. However, 15 of these cases needed an inotropic support of 10 mcg/kg/min for 24 hours and more. In two (7%) cases taken to surgery in emergency settings and with intra-aortic balloon support and in one case with a low ejection fraction, there was a need for combined inotropics (dopamine, dobutamine, adrenaline, and noradrenaline). This support was required for at least 72 hours. Mortality occurred in one (3%) case and this was a patient who was taken into an emergency bypass operation. When the relationship between the changes in hemodynamic parameters and IMA changes in the patients was examined, no statistical significance was identified (Mann-Whitney U, p > 0.05).

**Table 1:** Demographics characteristics of the cases

		%
***Age (years)***	58,6	

***Male, n (%)***	22	-73%

***Diabetes Mellitus, n***	11	-36%

***Hypertension, n***	15	-50%

***Smoking, n***	17	-56%

***Ejection Fraction, n***	12 cases 35 >	-40%
	7 cases 36-50	-23%
	11 cases 51 <	-37%

***Cardiopulmoner bypass time(Mean)***	81 min.	

***Cross clamp time(Mean)***	37 min.	

***Number of bypass vessel, n***	CABG x1...1	-3%
	CABG × 2...1	-3%
	CABG x3...11	-33%
	CABG x4...14	-42%
	CABG x5...3	-9%

***LIMA graft use, n***	27	-90%

## Discussion

Myocardial ischemia in various degrees may develop during the intraoperative phase due to such reasons as manipulation of the heart, inadequate myocardial protection, and incomplete revascularization. In order to reveal myocardial perfusion defect, such markers as AST, myoglobin, myocardial creatine kinase isoenzymes, and troponin have been used until today [12,17,18]. Recent studies show that the structure of serum albumin changes when ischemia develops in the body. Normal human albumin has the N terminal region, which detoxifies free oxygen radicals. The N region is also the area to which such transition metals as cobalt, copper and nickel are bound. In the ischemia condition, the N region is damaged by oxygen radicals. Albumin's capacity to bind metals such as nickel, cobalt and copper is diminished [7,14,19]. The resultant albumin as such is IMA. The type of the radical that affects the N terminal region most is the hydroxyl radical. Free radical binding capacity of IMA is very low. Elevation of IMA is directly associated with free radicals that form during ischemia [20]. IMA is also one of the markers of oxidative stress [21,22]. Reperfusion after ischemic conditions includes more free radical and iron and copper exposure, which may cause even more alterations to albumin than ischemia itself [23,24]. In studies conducted in earlier years, it was shown that IMA levels elevate in cardiac ischemia, cerebrovascular occlusion, pulmonary ischemia, gastrointestinal ischemia, and muscle ischemia [3,7,8,19,25-27].

A study conducted to scrutinize IMA specifies that IMA elevation may occur due to coronary vasospasm [5]. In other words, while the other markers for cardiac ischemia necessitate necrosis formation, IMA is elevated even in the early stages of ischemia. The most important characteristic that differentiates IMA from other cardiac ischemia markers is that it increases in the early phase particularly. It elevates in just minutes, peaks within 2 to 4 hours and returns to normal in 6 to 12 hours [3,28]. Non-existence of myocardial ischemia is considered to be confirmed in 90-95% of the cases if IMA levels are normal in the presence of a normal ECG and normal troponin levels [5]. Rapid elevation of IMA in minutes could render it usable in patient follow up and for taking necessary precautions rather earlier.

In this study, we examined the effects of coronary bypass operations on IMA levels and linkage of these levels with hemodynamic parameters. It was determined in a previous study that IMA levels increased shortly after the operation and decreased later on. However, it was stated that the amount of this decrease was still higher than basal levels [29]. In our study, we found that IMA levels elevated rapidly in the first 30 minutes following cross-clamping and decreased during the postoperative 6-hour-phase. However, the amount of this decrease did not reach to the level of the preoperative values. We believe that this can be attributed to a temporary effect created by reperfusion. In our study, IMA levels in the samples taken during the intraoperative stage were statistically higher than those obtained in the preoperative and postoperative periods.

Atrial fibrillation is observed in 7 to 40% of the cases after coronary bypass surgery [30,31]. Paroxysmal AF attacks emerged in 53% of our cases. The rate of atrial fibrillation that necessitated treatment for more than 72 hours was 20%.

One of the major factors that determines the need for inotropics during the postoperative period is the duration of cross-clamping. If the duration of cross-clamping is longer than 90 minutes, the need for inotropics increases [31]. In 23 (76%) of our cases, a need for an inotropic (7-15 microgram/kg/min dopamine) developed. Inotropic support of 10 mcg/kg/min for 24 hours and more became necessary in 15 (50%) of the cases that required an inotropic. Arrhythmia that did not require any need for treatment developed in 11 (36%) of the cases in the postoperative period. When the cases with prolonged inotropic need as well as the cases with atrial fibrillation who needed treatment for more than 72 hours (6 cases) were examined, it was understood that the postoperative IMA levels in these patients remained higher than the rest of the cases. Although intraoperative IMA levels were statistically significantly higher in all cases, it was found that especially in some cases, arrhythmias lasted longer and more need for inotropics was present. This finding may imply a suspected presence of a subgroup specific to myocardium. Ventricular arrhythmia, atrial fibrillation, and inotropic requirement were found to be more in patients whose IMA values were measured higher. While this excess was not statistically significant, it was proportionally higher (in terms of the % value). It might be possible to attain statistical significance by increasing the number of the patients. Comparing with the preoperative stage, IMA levels were elevated more in samples taken 6 hours after the operation. The reason for this could be a temporary ischemic condition caused by reperfusion. We believe that these levels would come closer to basal values in the samples to be taken in the upcoming hours. The most important factor that restricts the use of IMA is that it is not a specific marker for myocardium. That being said, IMA may increase in some conditions (septic shock, renal failure, neoplasia, etc.) [32].

## Conclusion

Many further studies are required in order to determine the role of IMA in ischemic conditions that occur in various organs. Sensitivity of cells to ischemia varies from one organ to another. It is important to know the optimal IMA levels to diagnose ischemia particularly in organs such as the heart and brain. Increasing the number of the cases and lengthening measurement periods in these types of studies might help establish this optimal level. IMA is one of the markers that show myocardial ischemia earliest. There is a parallel association between IMA levels and hemodynamic parameters of the patients. The most important factor that restricts IMA is that IMA may elevate in other pathologies as well, which brings about a requirement for subgroup studies. Since IMA is an early marker, measurement of IMA levels may contribute to patient follow up and initiation of treatment at early stages.

## List of abbreviations

IMA: ischemia modified albumin; CABG: coronary artery bypass grafting; LIMA: Left internal mammarian artery; LAD: Left anterior descending artery; AF: Atrial fibrillation; DTT: dithiothreitol; ABSU: Absorbance Units; AST: aspartate aminotransferase.

## Competing interests

The authors declare that they have no competing interests.

## Authors' contributions

MK conceived of the study, participated in its design and coordination. and drafted the manuscript. SY participated in the design of the study and performed the statistical analysis. All authors have read and approved the final manuscript.
